# Expression changes and clinical significance of serum neuron-specific enolase and squamous cell carcinoma antigen in lung cancer patients after radiotherapy

**DOI:** 10.1016/j.clinsp.2022.100135

**Published:** 2023-03-24

**Authors:** Lulu Sun, Qing Shao

**Affiliations:** No.7 Departments of Oncology, The First People's Hospital of Lianyungang, Jiangsu, China

**Keywords:** Lung cancer, Radiotherapy, Neuron-specific enolase, Squamous cell carcinoma antigen, Clinical significance

## Abstract

**Objective:**

To explore the changes and clinical significance of serum Neuron-Specific Enolase (NSE) and Squamous Cell Carcinoma antigen (SCC) in patients with lung cancer before and after radiotherapy.

**Methods:**

82 patients with lung cancer were treated with radiotherapy, and effective clinical intervention was given during the radiotherapy process. The patients were followed up for 1 year after radiotherapy and were divided into a recurrence and metastasis group (*n* = 28) and a non-recurrence and metastasis group (*n* = 54) according to their prognosis. Another 54 healthy volunteers examined in the present study's hospital during the same period were selected as the control group. To compare the changes of NSE and SCC levels in serum in patients with lung cancer at admission and after radiotherapy, and to explore their clinical significance.

**Results:**

After intervention, NSE and SCC levels in the serum of the two groups of patients were significantly lower than those before intervention, and the levels of CD4^+^ and CD4^+^/CD8^+^ were significantly higher than those before intervention (*p* < 0.05); the level of CD8^+^ was not significantly different from that before intervention (p > 0.05). And NSE and SCC levels in the intervention group were significantly lower than those in the routine group, the levels of CD4^+^, CD4^+^/CD8^+^ were significantly higher than those in the routine group (*p* < 0.05).

**Conclusion:**

NSE and SCC in serum can preliminarily evaluate the effect of radiotherapy in patients with lung cancer and have a certain predictive effect on prognosis.

## Highlights

• The aim is to explore NSE and SCC in patients with lung cancer before and after radiotherapy.
• Total of 82 patients with lung cancer were selected as the study group.
• NSE and SCC levels can preliminarily evaluate the effect of radiotherapy.

## Introduction

Lung cancer is one of the most common malignant tumors of the respiratory system, and its morbidity and mortality rank first among all malignant tumors, becoming a global public health problem.^1^^,^^2^ At this stage, surgery, radiotherapy, and chemotherapy are effective methods for the treatment of lung cancer, but most patients have developed into the middle and late stages when they were first diagnosed, thus losing the best time for surgery, making radiotherapy and chemotherapy the main treatment methods for lung cancer.^3^ However, chemotherapy has serious side effects, so radiotherapy has become a commonly used clinical treatment method.^4^ The poor prognosis of lung cancer patients after treatment is the main cause of death of patients, and the effect of radiotherapy will directly affect the prognosis of patients. Therefore, it is of great significance to seek relevant serum indicators to effectively predict the effect of radiotherapy for clinically taking corresponding prognostic treatment measures. Neuron-Specific Enolase (NSE) is an enzyme secreted by tumors originating from neuroendocrine tissues, especially overexpressed in small-cell lung cancer. It is the most sensitive and specific tumor marker for Small Cell Lung Cancer (SCLC).^5^ Squamous Cell Carcinoma Antigen (SCC) was originally extracted from cervical squamous cell carcinoma in 1977 and is a sub-fragment of tumor-associated antigen TA-4, widely presenting in the cytoplasm of squamous cell carcinomas of the uterus, cervix, lung, head and neck, etc., especially in non-keratinizing carcinoma cells, the content is more abundant.^6^ Related studies have shown that NSE and SCC in serum can predict the effect of chemotherapy in patients with lung cancer.^7^^,^^8^ However, there is still a lack of relevant research on the changes and clinical significance of NSE and SCC in serum in patients with lung cancer before and after radiotherapy. Therefore, the levels of NSE and SCC in serum in patients with lung cancer before and after radiotherapy were monitored and their clinical significance was discussed in this study.

## Materials and methods

### Study population

Lung cancer patients admitted to the present study's hospital from April 2020 to March 2021 were selected as the research subjects. The inclusion criteria were as follows: (1) All patients met the World Health Organization's diagnostic criteria for lung cancer and were diagnosed by imaging and pathological analysis; (2) The Tumor Node Metastasis (TNM) staging ranges from IIb to III; (3) With radiotherapy indications; (4) Underwent radiotherapy with the same regimen; (5) The survival period was more than 3 months; (6) The patients and their families were informed and agreed. The exclusion criteria were as follows: (1) Physically intolerant; (2) Combined with other malignant tumors; (3) With severe heart, liver, kidney, and other important organ dysfunction; (4) Pregnant and lactating women. According to the above inclusion and exclusion criteria, a total of 82 patients were included in this study, including 50 males and 32 females; age ranges from 37 to 73 years old, with an average of (55.08±9.14) years old; TNM staging: 45 cases of stage II and 37 cases of stage III; tumor types: adenocarcinoma in 35 cases, squamous cell carcinoma in 29 cases, and small cell carcinoma in 18 cases. Another 54 healthy volunteers examined in the hospital during the same period were selected as the control group, with 32 males and 22 females, with age ranges from 35 to 74 years old, with an average age of (54.88±9.76) years old. The control group was informed and voluntarily participated in the research. There was no significant difference in general data such as age and gender between the two groups (p > 0.05), which was comparable.

### Nursing method

A total of 82 patients were randomly divided into an intervention group and routine group, with 41 cases in each group. The routine group received routine clinical care, the main contents included health knowledge education before radiotherapy, close monitoring of the patient's condition, and a guide for treatment preparation. Among them, health education promotion includes basic knowledge of lung cancer, treatment methods, precautions for radiotherapy, possible adverse reactions, etc.

The intervention group was given comprehensive clinical intervention: 24 hours after the patient was admitted to the hospital, the nursing staff explained the department system and specific situation to the patient and their family members, introduced the hospital and ward environment to the patient, and eliminated the discomfort of the patient to the unfamiliar environment. Professional nurses carry out health knowledge education for patients, including the pathogenesis of lung cancer, influencing factors, the necessity of radiotherapy, the purpose of radiotherapy, expected effects, and possible adverse reactions during radiotherapy, and teach patients emotional management content. Additionally, health education manuals are distributed to patients, so that patients can fully understand the contents of lung cancer and treatment and make them aware of the importance of radiotherapy. Apart from this, the patient's family background and social background were investigated in order to better understand the patient's cognition of the disease, and the targeted health education was carried out according to the cognition of different patients in nursing practice to strengthen patients' understanding of radiotherapy. During radiotherapy, nurses closely monitored the patient's condition, paid attention to the patient's psychological dynamics, corrected cognitive errors in patients during radiotherapy, and guided patients to actively and objectively evaluate their own state. For the nervousness, anxiety, fear and other negative emotions of patients, timely and effective psychological counseling was carried out to strengthen the emotional management of patients. At the same time, during radiotherapy, scientific and reasonable dietary plans were formulated for patients. For example, nutritional intervention can be performed if necessary for patients with poor appetite, and symptomatic treatment such as antiemetic treatment should be given to patients with severe vomiting. Furthermore, according to the actual situation, the patients were given anti-infection, anti-infection, sleep aid, and other symptomatic treatments. Moreover, ward nursing should be done well during radiotherapy, including basic temperature and humidity control, timely disinfection, etc., and keeping the room quiet.

### Measurement of NSE and SCC

At the time of admission and after radiotherapy, the cubital vein blood (3 mL) was drawn from patients in the study group. After centrifugation at 2000 r/min for 10 min, the upper serum was collected, and NSE and SCC levels in the serum of the patients were measured by Enzyme-Linked Immunosorbent Assay (ELISA). The NSE and SCC kits were purchased from Wuhan Fein Biotechnology Co., Ltd., and the operation process was carried out in strict accordance with the kit instructions.

### Measurement of T lymphocyte subsets

Before and after the intervention, about 3 mL of cubital vein blood was drawn from the patients in the study group. After anticoagulation treatment, a monoclonal antibody was added and incubated at room temperature in the dark for 20 min, then red blood cell lysate was added, and the cells were protected from light for 15 min. After centrifugation at 2000 r/min for 5 min, the supernatant was discarded, and PBS buffer was added to mix well. Finally, the number of T lymphocyte subsets was determined by a cell analyzer (BD FACSCanto, USA).

### Follow-up

The patients in the study group were followed up by telephone for 1 year after discharge, and the follow-up time was until March 31, 2022. According to tumor recurrence and metastasis, they were divided into the recurrence and metastasis group (*n* = 28) and the non-recurrence and metastasis group (*n* = 54).

### Statistical analysis

The research data were imported into SPSS 22.0 software for statistical analysis. The levels of serum NSE, SCC and T lymphocyte subsets were expressed by mean ± standard deviation, and the comparison was performed by Student's *t*-test. The Receiver Operating Curve (ROC) was used to evaluate the predictive efficacy of NSE and SCC levels in serum after radiotherapy on the prognosis of patients. A difference was considered significant when the p-value was less than 0.05.

## Results

### Comparison of NSE and SCC levels in serum between the control group and research group at admission

At admission, the NSE and SCC levels in serum in the study group were significantly higher than those in the control group, and the difference was statistically significant (*p* < 0.001), as shown in Table 1.

**Table 1:** The NSE and SCC levels in serum between the control group and study group.

Groups	NSE (ng/mL)	SCC (ng/mL)
Study group (*n* = 82)	37.66 ± 9.25	1.72 ± 0.24
Control group (*n* = 54)	10.14 ± 2.72	0.67 ± 0.12
*t*	21.242	29.766
p	*p* < 0.001	*p* < 0.001

### Changes of NSE and SCC levels in serum in the study group before and after radiotherapy

After radiotherapy, the NSE and SCC levels in the serum of the patients were significantly lower than those before radiotherapy (*p* < 0.001), as shown in Table 2.

**Table 2:** The NSE and SCC levels in serum changes in the study group.

Time	NSE (ng/mL)	SCC (ng/mL)
Before radiotherapy (*n* = 82)	37.66 ± 9.25	1.72 ± 0.24
After radiotherapy (*n* = 82)	25.33 ± 7.47	1.56 ± 0.35
*t*	9.391	3.414
p	*p* < 0.001	*p* < 0.001

### Comparison of NSE and SCC levels in serum in patients with different prognoses

After radiotherapy, NSE and SCC levels in serum in the recurrence and metastasis group were significantly higher than those in the non-recurrence and metastasis group (p = 0.013, 0.008), as seen in Table 3.

**Table 3:** Comparison of NSE and SCC levels in serum in patients with different prognosis.

Prognosis	NSE (ng/mL)	SCC (ng/mL)
Recurrence and metastasis (*n* = 28)	27.32 ± 5.36	1.71 ± 0.32
Non-recurrence and metastasis (*n* = 54)	24.30 ± 4.97	1.48 ± 0.38
*t*	2.540	2.737
p	0.013	0.008

### Predictive efficacy of NSE and SCC in serum on tumor recurrence and metastasis in lung cancer radiotherapy patients

**Fig. 1:**
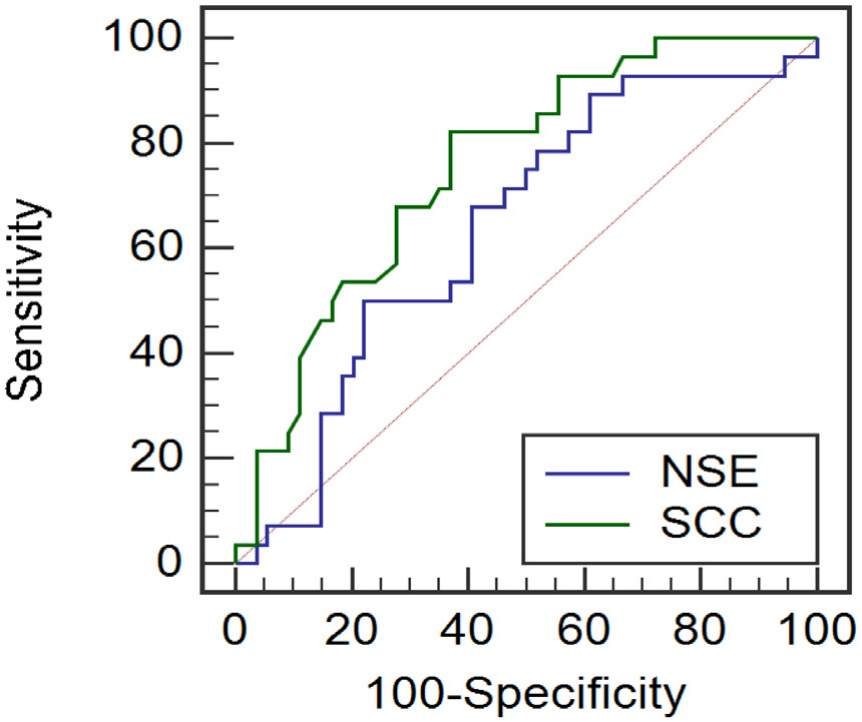
ROC curves of NSE and SCC in serum on tumor recurrence and metastasis in lung cancer radiotherapy patients.

The ROC curve analysis showed that the AUC of NSE and SCC levels in serum for predicting tumor recurrence and metastasis in patients with lung cancer radiotherapy was 0.848 and 0.755, respectively, the critical values were 21.98 ng/mL and 1.59 ng/mL, and the sensitivity was 92.86% and 82.14%, and the specificity was 82.14%, 71.87%, 62.96%, as exhibited in Fig. 1.

### Comparison of NSE, SCC and T lymphocyte subsets levels in serum before and after intervention

After the intervention, the NSE and SCC levels in the serum of the two groups were significantly lower than those before intervention (*p* < 0.05), the levels of CD4^+^ and CD4^+^/CD8^+^ were obviously higher than those before intervention (*p* < 0.05); the CD8^+^ level was not significantly different from that before the intervention (p > 0.05); The levels of serum NSE and SCC in the intervention group were significantly lower than those in the routine group, and the levels of CD4^+^ and CD4^+^/CD8^+^ were significantly higher than those in the routine group (*p* < 0.05); there was no significant difference in CD8^+^ levels between the two groups compared with the routine group (p > 0.05), as seen from Table 4.

**Table 4:** Comparison of NSE, SCC and T lymphocyte subsets levels in serum before and after intervention.

Index	Intervention group (*n* = 41)		Routine group (*n* = 41)	
	Before intervention	After intervention	Before intervention	After intervention
CD4^+^ (%)	28.21 ± 5.24	34.67 ± 5.77^a^^,^^b^	28.55 ± 5.34	31.46 ± 6.37^a^
CD8^+^ (%)	24.67 ± 5.77	24.16 ± 5.17	24.88 ± 6.26	25.04 ± 7.71
CD4^+^/CD8^+^	1.14 ± 0.13	1.43 ± 0.22^a^^,^^b^	1.15 ± 0.11	1.26 ± 0.24^a^
NSE (ng/mL)	38.66 ± 8.25	27.54 ± 5.42^a^^,^^b^	37.54 ± 9.26	31.24 ± 7.74^a^
SCC (ng/mL)	1.74 ± 0.24	1.48 ± 0.24^a^^,^^b^	1.76 ± 0.34	1.62 ± 0.21^a^

Note: Compared with the same group before intervention.

a *p* < 0.05; compared with conventional group after intervention,

b *p* < 0.05.

## Discussion

Lung cancer is one of the malignant tumors that threaten human life. Although radiotherapy and chemotherapy of lung cancer have made breakthroughs in recent years, which can prolong the life cycle of patients to a certain extent, there is still a high risk of recurrence and metastasis after treatment, which is the main reason for the poor prognosis.^9^^,^^10^ In the occurrence and development process of tumor cells, a series of tumor markers will be synthesized or released. The existence or quantitative changes of such markers can effectively reflect the occurrence, cell differentiation, and function of tumors, so as to provide basis and guidance for clinical diagnosis and effective treatment of tumors, as well as the further prediction of prognosis.^11^^,^^12^ NSE is enolase involved in the glycolytic pathway, mainly found in neural and neuroendocrine tissues. Wang's team found that NSE is useful for predicting liver metastasis of lung cancer.^13^ Bai's team found that NSE has a certain value in judging the prognosis of lung cancer patients.^14^ SCC is a squamous cell carcinoma antigen, its main component is a glycoprotein, which belongs to the tumor-associated antigen TA-4 subtype. SCC mainly exists in squamous cell carcinoma, with the most abundant content in lung cancer and uterine cancer.^15^ Yang^16^ research group found that SCC has certain clinical value for the diagnosis of lung cancer. The Cho^17^ research group found that SCC is closely related to the prognosis of lung cancer patients. Based on the above research, this study evaluated the changes in NSE and SCC levels in patients with lung cancer before and after radiotherapy, in order to more accurately evaluate the effect of radiotherapy and provide theoretical guidance for prognosis evaluation and further treatment.

The results showed that the levels of serum NSE and SCC in the study group were significantly higher than those in the control group, suggesting that NSE and SCC were related to the occurrence of lung cancer, which was consistent with the results of previous studies.^18^, ^19^ The results showed that the levels of NSE and SCC decreased significantly after radiotherapy, suggesting that radiotherapy can improve the levels of NSE and SCC, which may be attributed to the killing and inhibitory effects of radiotherapy on tumors, further reducing the malignancy of tumors. The results showed that the levels of NSE and SCC in the recurrence and metastasis group were significantly higher than those in the non-recurrence and metastasis group, indicating that the levels of NSE and SCC were closely related to tumor recurrence and metastasis. It is speculated that the tumor metabolism level in lung cancer patients with recurrence and metastasis is high, which can promote the combination and release of NSE and SCC. Further ROC curve analysis showed that the AUC of NSE and SCC levels for predicting tumor recurrence and metastasis in Lung cancer radiotherapy patients was 0.848 and 0.755, the critical value was 21.98 ng/mL and 1.59 ng/mL, the sensitivity was 92.86% and 82.14%, and the specificity was 71.87% and 62.96%, respectively. The above results suggested that NSE and SCC levels in serum can initially predict the prognosis of patients with lung cancer radiotherapy patients. However, the specificity of SCC is low, other indicators can be considered for comprehensive prediction in the later stage.

In this study, lung cancer patients were treated with routine nursing care and comprehensive clinical intervention. The results showed that after the intervention, the NSE and SCC levels in the serum of the two groups of patients were significantly lower than those before the intervention, and the levels of CD4^+^ and CD4^+^/CD8^+^ were apparently higher than those before the intervention; CD8^+^ level was not significantly different from that before intervention. Besides, the NSE and SCC levels in serum in the intervention group were significantly lower than those in the conventional group, the levels of CD4^+^ and CD4^+^/CD8^+^ were obviously higher than those in the conventional group. There was no apparent difference in CD8^+^ levels between the two groups. The above results show that effective clinical intervention can improve the NSE and SCC levels in serum in lung cancer patients and reduce the inhibitory effect of radiotherapy on the immune function of the body to a certain extent.

In conclusion, NSE and SCC levels in serum can effectively reflect the radiotherapy effect of lung cancer patients and show a certain predictive value for the prognosis effect after radiotherapy, providing information for the clinical radiotherapy effect and prognosis, and the basis and guidance for further clinical treatment. Nevertheless, the detection of a single indicator may be insufficient, and the combined detection of multiple indicators should be considered in actual clinical practice. Importantly, effective clinical intervention for radiotherapy patients can effectively enhance the therapeutic effect and alleviate the immunosuppression caused by radiotherapy.

## Ethics Committee Statement

The authors have no Clinical Trial registration number because According to the regulations of our affiliation, the present research only needs the approval of our own affiliation to conduct research, but the study was approved by the ethics committee of The First People's Hospital of Lianyungang (n° 20200306).

## Authors’ contributions

Substantial contributions to the conception or design of the work; or the acquisition, analysis, or interpretation of data for the work: Lulu Sun, Qing Shao.

Drafting the work or revising it critically for important intellectual content: Lulu Sun, Qing Shao.

Final approval of the version to be published: Lulu Sun, Qing Shao.

## Conflicts of interest

The authors declare no conflicts of interest.
